# An audit of high-dose and combination antipsychotic prescribing across the general adult inpatient wards in Mersey Care NHS Foundation Trust

**DOI:** 10.1192/bjo.2021.257

**Published:** 2021-06-18

**Authors:** Declan Hyland, Beth Hemmings, Yasmine Elagamy

**Affiliations:** 1 Consultant Psychiatrist, Clock View Hospital, Liverpool, Mersey Care NHS Foundation Trust; 2 5th year medical undergraduate, University of Liverpool; 3 Core Trainee 1 in Psychiatry, Clock View Hospital, Liverpool, Mersey Care NHS Foundation Trust

## Abstract

**Aims:**

To review the number of prescriptions of regular high-dose antipsychotics and combination antipsychotic therapy across the eight general adult inpatient wards in Mersey Care NHS Foundation Trust and examine whether these prescriptions followed Trust recommendations for high-dose antipsychotic therapy (HDAT).

**Background:**

The two main rationales behind prescribing HDAT are pharmacokinetics differ in individuals and so insufficient amounts of antipsychotic may reach the effect site at maximum dose in some patients and variations in the effect site between patients may mean higher doses are required to achieve therapeutic effect.

**Method:**

The electronic prescription records for all patients on the eight general adult inpatient wards were scrutinised. 121 patients were prescribed antipsychotic medication. Any patients on a combination of regular antipsychotic medication or on HDAT were identified. Any patient on combination therapy or HDAT was studied to determine if Clozapine had been considered. The electronic notes of HDAT patients were analysed to ascertain whether tests recommended by Trust guidelines – BMI, blood pressure (B.P), pulse rate, ECG, FBC, U and Es, LFTs, serum prolactin, serum cholesterol and HbA1c level had been performed prior to initiation and following any dose increase.

**Result:**

21 of 121 patients prescribed antipsychotic medication were on combination therapy. 11 were subject to HDAT. 8 of the 11 HDAT patients were on combination therapy. Clozapine was considered before initiating HDAT in 9 of the HDAT patients. Clozapine was considered in 13 of the 21 patients on combination antipsychotic therapy, but only two were initiated on Clozapine (combined with Olanzapine or Risperidone).

100% of HDAT patients had an ECG prior to initiation of HDAT; only 36% had one after dose increases above BNF maximum. 100% of HDAT patients had their BMI measured before initiation. 91% had baseline B.P and heart rate checked. Of the recommended blood tests, 100% of HDAT patients had baseline FBC, U and Es, LFTs and serum cholesterol. Fewer patients had a baseline HbA1c level (91%) or serum prolactin (46%) measured.

**Conclusion:**

Prevalence of HDAT across the general adult inpatient wards in the Trust was 9%, much lower than the 28% reported in the HDAT audit completed by the Prescribing Observatory for Mental Health in 2012. Patients within Mersey Care are more likely to be prescribed combination therapy than HDAT. Not every HDAT patient has been considered for Clozapine. There is a need to ensure Trust monitoring guidelines for HDAT patients are being strictly adhered to.

